# Piezoresistivity in single DNA molecules

**DOI:** 10.1038/ncomms9032

**Published:** 2015-09-04

**Authors:** Christopher Bruot, Julio L. Palma, Limin Xiang, Vladimiro Mujica, Mark A. Ratner, Nongjian Tao

**Affiliations:** 1 Center for Bioelectronics and Biosensors, Biodesign Institute, School of Electrical, Computer, and Energy Engineering, Arizona State University, Tempe, Arizona 85287-5801, USA; 2 Department of Chemistry, Arizona State University, Tempe, Arizona 85287-5801, USA; 3 Department of Chemistry, Northwestern University, 1145 Sheridan Road, Evanston, Illinois 60208-3113, USA

## Abstract

Piezoresistivity is a fundamental property of materials that has found many device applications. Here we report piezoresistivity in double helical DNA molecules. By studying the dependence of molecular conductance and piezoresistivity of single DNA molecules with different sequences and lengths, and performing molecular orbital calculations, we show that the piezoresistivity of DNA is caused by force-induced changes in the π–π electronic coupling between neighbouring bases, and in the activation energy of hole hopping. We describe the results in terms of thermal activated hopping model together with the ladder-based mechanical model for DNA proposed by de Gennes.

## Abstract (web summary)

Piezoresistivity finds many applications in micro-electromechanical systems, but a piezoresistive material at a molecular level has not yet been demonstrated. Here, Bruot *et al*. show this effect in double helix DNA molecules due to the electronic coupling between neighbouring bases upon mechanical force.

## Graphical abstract

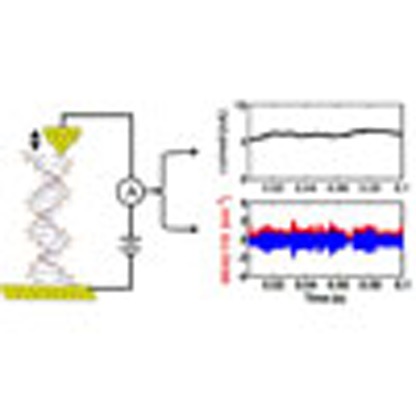

A mechanical force can induce a change in the resistivity of materials by distorting the interatomic spacing, and thus the electronic bandgap of the materials. This important property of materials, known as piezoresistivity, has many applications, including sensors and micro-electromechanical systems (for example, accelerometers). Similar piezoresistivity-like behaviours have been observed in single molecule devices, single molecules covalently bridged between two electrodes^1^,^2^,^3^,^4^. However, these behaviours arise primarily from molecule–electrode coupling effects, as opposed to distortions within the molecule causing changes of the molecular electronic states. Furthermore, to date, there have been no investigations into the piezoresistivity of more complex molecules such as DNA, and the role that distortions of the nucleic acid units play in charge transport.

Studying piezoresistivity in DNA also helps understand charge transport in DNA, a topic that has received persistent interest over the past two decades for its role in biological processes^5^,^6^ and potential device applications of DNA^7^,^8^. Strong evidence has shown that charge transport in DNA is mediated by the π–π stacking interaction between neighbouring bases^9^,^10^,^11^,^12^,^13^,^14^,^15^. Furthermore, experiments and theory have shown that intra-strand stacked guanines, sequences with neighbouring guanines on the same strand, have the lowest ionization energy among single or stacked bases, and contribute most significantly to charge transport in DNA^16^,^17^. For example, we recently showed, in Xiang *et al*., that designing DNA sequences with intra-strand guanine stacking increases the π–π interaction between hopping sites, causing delocalization of the hopping site over several bases that is observed as a coherent transport component of the thermally activated hopping regime^18^. With the important role that π–π coupling plays in DNA charge transport, one would expect that this transport is sensitive to the distance between neighbouring bases, leading to piezoresistivity in DNA.

Here we show that piezoresistivity analogous to that in bulk materials occurs as an intrinsic property of single DNA molecules, where conduction is due to thermally activated hopping of holes via guanine sites. By studying the sequence and molecular length dependence of DNA piezoresistivity, in combination with theoretical calculations, we conclude that the piezoresistivity is due to force-induced changes in both the hopping rate between sites and the activation energy for transport.

## Results

### Experimental setup

**Figure 1:**
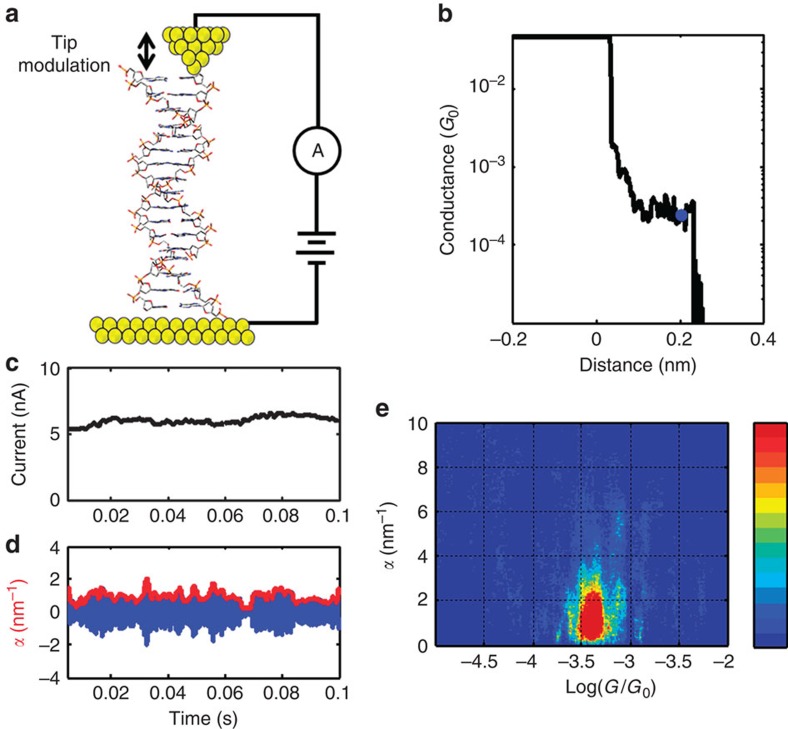
Experimental setup. (**a**) Schematic diagram of STM-BJ with a modulating tip. (**b**) Conductance versus distance decay curve for a single **G**–**C** molecule, where the blue dot marks the position at which the molecular conductance and piezoresistance are measured. (**c**) Low-frequency component of the current collected from the single double-stranded DNA junction shown in **b**, which gives the conductance of the molecule. (**d**) Red curve: amplitude of conductance modulation normalized with conductance, which describes the piezoresistivity in DNA (*α*). Blue curve: conductance modulation due to tip modulation. (**e**) *α* versus conductance histogram for **G**–**C** sequence.

To measure conductance and piezoresistivity in single DNA molecules, we use a tip modulation scanning tunnelling microscope break junction (STM-BJ) technique^19^. The STM-BJ technique has been used widely for more than a decade to measure charge transport in single DNA molecules. For example, STM-BJ measurements have shown that charge transport in single DNA molecules is mediated by thermally activated hopping between neighbouring guanine bases^9^. Further investigation of the mechanical stability of DNA under STM-BJ measurements showed that stretching single DNA molecular devices results in mechanical distortions of the DNA molecule, with the end bases distorted most until the DNA molecule eventually ruptures^20^. The basic idea of the tip modulated STM-BJ technique used in this report is described in Fig. 1. During traditional STM-BJ measurements, an STM tip is brought into contact with the target molecule, which is functionalized to a conducting surface. The junction conductance is then monitored while the tip is retracted until the molecular junction breaks and the conductance drops to zero. In the present technique, when a molecular plateau is detected (Fig. 1b), the tip retraction is stopped and a 0.02 nm sinusoidal modulation (1 kHz frequency) is applied to the tip position along the axis of the tip. The tip–substrate current of the mechanically modulated junction is then recorded for a 100-ms period, the molecular conductance is checked, and the current is either recorded for another 100 ms if the junction is undisturbed or the break junction process is repeated to form another molecular junction. This way, several hundreds of molecular junctions were collected for each DNA molecule studied here.

The conductance measured with the STM-BJ technique can be expressed with respect to the sinusoidal modulation (*A*_0_ cos(*ωt*)) as^19^


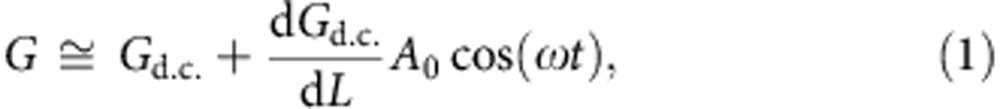


where *G*_d.c._ is the low-frequency portion of the conductance unaffected by modulation, *A*_0_ is the tip modulation amplitude and *ω* is the tip modulation frequency. The conductance thus has two components, a low-frequency component, *G*_d.c._, which is the molecular conductance (first term in equation (1) and Fig. 1c) and a component at the tip modulation frequency, 
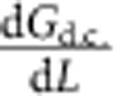
, which is proportional to the change in conductance with modulation (second term in equation (1)). A convenient way to describe piezoresistance in single DNA molecules is to normalize the amplitude at the modulation frequency by the molecular conductance, or *α*=|(1/*G*_d.c._)(d*G*_d.c._/d*L*)| (red curve in Fig. 1d). The Taylor expansion in equation (1) assumes weak dependence of conductance on small amplitude modulation, and is applicable to molecular electronics regardless of the transport mechanism.

Information collected through the tip modulated STM-BJ, the conductance (*G*_d.c._) and piezoresistance (*α*), for each molecule studied was then formed into two-dimensional (2D) *α* versus *G*_d.c._ histograms, each containing the conductance and piezoresistance measured from several hundred individual molecular junctions (Fig. 1e). The 2D histograms for each molecule studied are shown in the Supplementary Figs 2 and 3. Due to the broad peak widths common in single molecule measurements, a more convenient way of comparing the conductance and piezoresistance values between different DNA sequences is to compile the values of the 2D histogram onto the conductance and piezoresistance axes, respectively. This way the most probable value of conductance and *α* can be extracted from fitting the peak position in the corresponding histograms.

### DNA sequences

**Figure 2:**
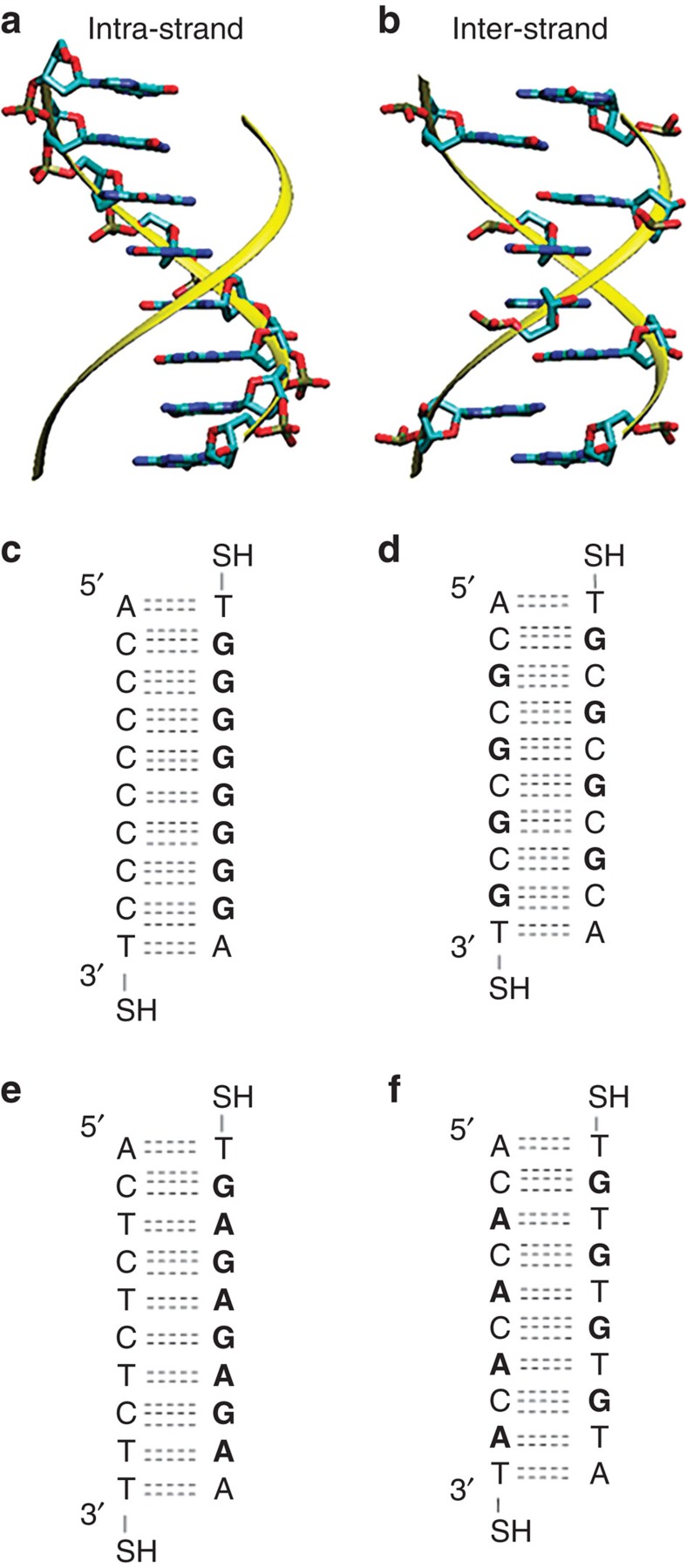
Molecular structures. Intra-strand purine (**a**) and inter-stand purine (**b**) stacked sequences, illustrating the spatial separation of purine bases. Intra-strand stacked sequences: **G**–**G** (**c**) and **G**–**A** (**e**) and inter-strand stacked sequences: **G**–**C** (**d**) and **G**–**T** (**f**).

To understand the role of π–π interaction and base proximity, we studied the conductance and piezoresistance of DNA molecules with different sequences and lengths. These molecules are terminated by a thymine and adenine base on the 3′ and 5′ ends, respectively. The remaining base pairs were chosen to have either inter-strand purines or intra-strand purines. Intra-strand purine sequences consist of a guanine base (**G**) neighboured by either another guanine or an adenine base (**G**–**G** and **G**–**A** respectively), putting neighbouring hopping sites on the same strand. Inter-strand purine sequences have cytosine or thymine bases neighbouring guanine bases on a single strand (**G**–**C** and **G**–**T**, respectively), so that nearest purine hopping sites are on different strands. The structures of these molecules are illustrated in Fig. 2a–f. The length dependence of molecular conductance and piezoresistance was measured for DNA ranging from 8 base pairs (∼2.7 nm) to 14 base pairs (∼4.7 nm) in length. Again, inter-strand and intra-strand sequences were investigated, although the sequences were designed to be self-complementary. In this way, the inter-strand purine sequences has alternating guanine and cytosine on a single strand and the intra-strand purine sequence is a self-complementary sequence with guanine bases neighbouring each other, called (**G**–**C**)_*N*_ and (**G**_*N*_–**C**_*N*_), respectively. To confirm B-form DNA structure for the molecules studied circular dichroism, polyacrylamide gel electrophoresis and melting temperature experiments were carried out under the same conditions as STM-BJ measurements, see Supplementary Figs 7–9 and Supplementary Note 3.

### DNA conductance and piezoresistance

**Figure 3:**
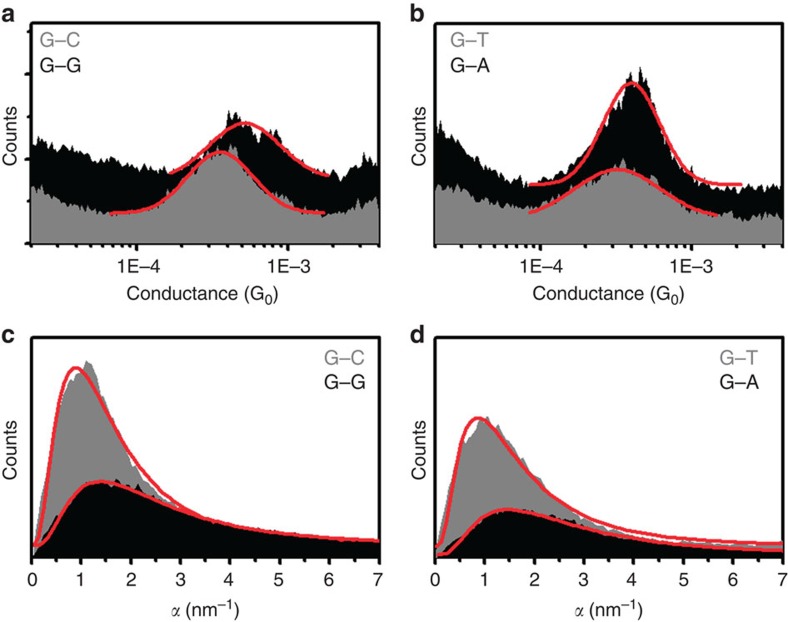
Conductance and piezoresistance of DNA with different sequences. (**a**) Conductance histograms comparing **G**–**C** (grey) with **G**–**G** (black). (**b**) Conductance histograms comparing **G**–**T** (grey) with **G**–**A** (black). Red lines indicate Gaussian fittings. (**c**) *α* histograms comparing **G**–**C** (grey) with **G**–**G** (black). (**d**) *α* histograms comparing **G**–**T** (grey) with **G**–**A** (black). The peak position for intra-strand purine sequences (**G**–**G** and **G**–**A**) are larger than for inter-strand purine sequences (**G**–**C** and **G**–**T**). Red lines indicate log-normal function fitting. Grey conductance histograms are offset for clarity.

The sequence dependence of conductance and piezoresistance are shown in Fig. 3. We find that the most probable conductance values for the intra-strand purine stacked sequences are larger than those for the inter-strand purine sequences, as shown in Fig. 3a,b, respectively. Specifically, log-scale histograms indicate that the **G**–**G** sequence has a conductance that is ∼1.3 times larger than that of **G**–**C** and the **G**–**A** sequence molecular conductance is ∼1.4 times larger than the conductance of **G**–**T**. This is an interesting finding, considering the constituent bases do not change between the **G**–**G** and **G**–**C** molecules, likewise for **G**–**A** and **G**–**T**. A list of conductance and piezoresistance values is shown in Supplementary Table 1 and linear conductance histograms are shown in Supplementary Fig. 4. As was previously shown^18^, this can be understood as a manifestation of the increase in π–π overlap between hopping sites for intra-strand purine stacks over inter-strand purine neighbours. To show this further, we calculated electronic couplings (V) between neighbouring base pairs for intra-strand and inter-strand sequences using the INDO/S method^21^. We find that the effective value of the couplings along the sequences are: *V*_GG_=0.141 eV, *V*_GC_=0.052 eV, *V*_GA_=0.098 eV, *V*_GT_=0.012 eV, in qualitative agreement with the experimental trend, since at the thermally activated hopping level, the electronic coupling is the key parameter that determines the difference on the conductance between different sequences^22^, in contrast with previous approaches based on the super exchange model^23^ (calculation details are presented in methods and Supplementary Note 4 and a full list of couplings is in Supplementary Table 2).

A significance difference is also seen in the piezoresistance, *α*, for the different sequences. As shown in Fig. 3c,d, the piezoresistance for intra-strand purine sequences is ∼1.5 times larger than that of the respective inter-strand purine sequences. This indicates that the conductance of intra-strand purine sequences is more sensitive to mechanical modulation than the inter-strand purine interactions. The observation of higher molecular conductance and piezoresistance for intra-strand purine stacked sequences suggests that the π–π interaction between neighbouring guanine hopping sites plays an important role in charge transport and sensitivity to mechanical perturbation in DNA molecules.

**Figure 4:**
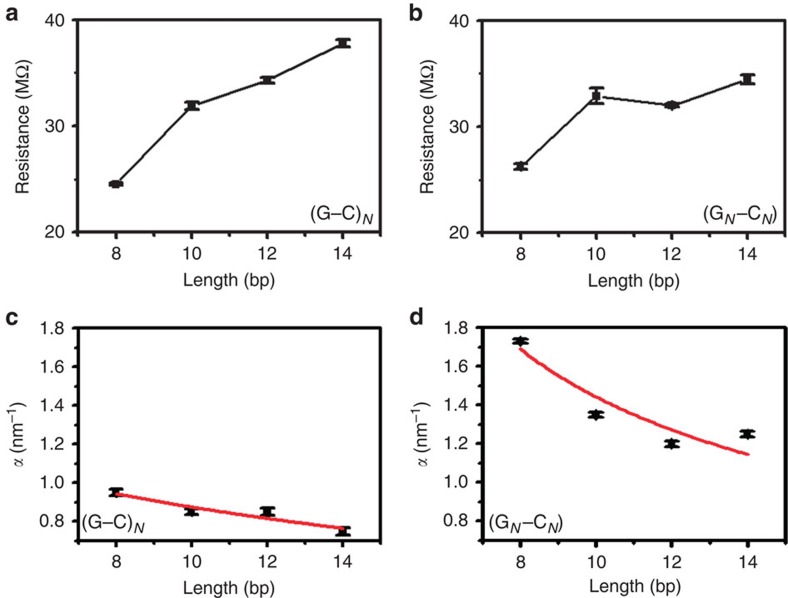
Length dependence of conductance and *α*. (**a**,**b**) Resistance versus molecular length for (**G**–**C**)_*N*_ and (**G**_*N*_–**C**_*N*_) sequences, respectively. Both molecular sequences are approximately linearly (as opposed to exponentially) dependent on molecular length. Note, however, that oscillations in resistance do occur, particularly for the intra-strand purine stacked sequence (**G**_*N*_–**C**_*N*_), see discussion in Supplementary Information. (**c**,**d**) *α* versus molecular length for (**G**–**C**)_*N*_ and (**G**_*N*_–**C**_*N*_) sequences, respectively. Red lines are fitted using the thermally activated hopping model with de Gennes' stretching correction. Note that the intra-strand purine stacked sequence (**G**_*N*_–**C**_*N*_) is larger and more sensitive to molecular length than the inter-strand purine sequence (**G**–**C**)_*N*_. Error bars represent the fitting error for histograms fit with Gaussian peaks.

To further understand the effect of π–π stacking between neighbouring bases on the piezoresistance, it is importance to investigate the dependence of both the conductance and piezoresistance on molecular length. Figure 4 shows the dependence of DNA conductance and *α* on molecular length. The DNA resistance (inverse of conductance) is not exponentially dependent on length, as in tunnelling transport, but instead, the DNA resistance is roughly linearly dependent on length as is expect for transport in the thermally activated hopping regime (Fig. 4a,b). A close examination of the resistance versus length plots reveals a weak oscillatory behaviour, especially for the intra-strand guanine sequence, which is consistent with the findings of Xiang *et al*.^18^. Considering a transport model that is intermediate between thermally activated hopping and coherent transport (treated by Büttiker as a coherent correction to the incoherent transport regime^24^), we find a hopping site delocalization length of ∼2 base pairs for the intra-strand purine stacked sequence, in agreement with the previously reported value^18^ (see Supplementary Note 3 and Supplementary Fig. 5f or further details on fitting). The presence of delocalization of the hopping site in these sequences strengthens the claim of strong coupling between neighbouring guanine hopping sites of the intra-strand purine stacked sequence. For this series of DNA molecules, the conductance difference between intra-strand and inter-strand purine sequences is less pronounced than those in Fig. 3. This is likely due to a minor difference in the system where there is a breaking in the stacking involved in making the intra-strand sequences self-complimentary and the change in the end orientations.

The piezoresistance of intra-strand purine stacked sequences is consistently larger than the inter-strand purine sequences for all lengths measured, decreasing in magnitude for longer DNA (Fig. 4c,d). The observed length dependence of the DNA piezoresistance (*α*) is in contrast to a recent report by Rascon-Ramos *et al*.^4^, which shows that *α* increases with molecular length for alkane family molecules. They attributed the observation to a signature of mechanical perturbation being distributed primarily across the electrodes. A fundamental difference between the alkane family and double helical DNA molecules is that the former, consisting of linear covalent C–C bonds, is more rigid than Au–Au bond in the electrodes, and the latter, held together with the relatively weak hydrogen bonds and π–π stacking force, is much softer than the Au–Au bond. So, we expect that in the case of DNA, the mechanical stretching is primarily distributed along the soft double helical molecule, rather than across the electrodes. This conclusion is further supported by several facts. First, the spring constants of Au–Au bond, C–C bond and DNA (14 base pairs) are 8 N m^−1^ (refs 25, 26), 520 N m^−1^ (ref. 27), 0.2 N m^−1^ (refs 28, 29), respectively. Second, *α* in the case of DNA is sensitive to DNA sequence, with intra-strand purine significantly larger than the inter-strand purine sequences. Finally, our recent experiment shows that the mechanical breakdown properties of the amine- and thiol-terminated DNA molecules are similar^20^. On the basis of these considerations, we conclude that DNA is stretched most in the STM-BJ measurement, and *α* measures the piezoresistance of DNA, rather than the electrodes or the electrode–molecule contact. We provide a more quantitative analysis of the experimental data of DNA in terms of charge transport and mechanical models of DNA below.

### Piezoresistance theory

First, we consider the dependence of DNA charge transport on the distance between neighbouring bases. For DNA systems, the transport mechanism between the STM tip and substrate has been found to be the thermally activated hopping process with a correction arising the finite coherence^18^. The coherence correction is a relative small contribution for the DNA sequences studied here (see Fig. 4a,b and the discussion n Supplementary Note 3), and thus may be neglected for simplicity. This approximation allows us to use the model by Nitzan^30^, which expresses the resistance for a bridge of N hopping sites as


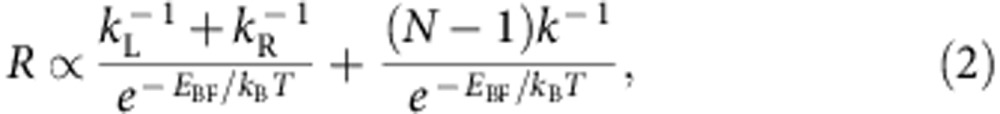


where *k*_B_ is Boltzmann's constant, *e* is the electron charge, *T* is the temperature, *E*_BF_ is the activation energy, *k*_L(R)_ is the transfer rate between the left (right) electrode and the molecule, and *k* is the transfer rate between neighbouring hopping sites. The terms that are dependent on the details of the molecular system are the activation energy, which is the difference between the electrode Fermi energy and the guanine highest occupied molecular orbital (HOMO) energy, and the transfer rate between neighbouring hopping sites.

As stated above, the piezoresistance is the normalized change in conductance with respect to tip modulation, or in terms of resistance *α*=|−(1/*G*_DC_)(d*R*_DC_/d*L*)|. The resistance for the thermally activated hopping model, equation (2), has two terms, which are dependent on hopping site distance modulation. The first is the transfer rate between neighbouring hopping sites, which depends exponentially on the separation between neighbouring bases, such that *k*≈*k*_0_exp(−*βz*). The other distance dependent term is the activation energy, *E*_BF_, which is related to the guanine hopping site energy. Taking these terms as depending on the change in hopping site distance, the piezoresistance can be expressed as:


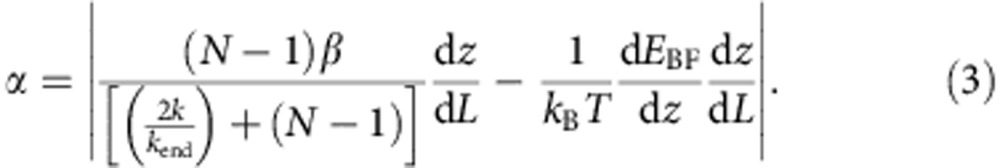


The first term in equation (3) depends on the decay constant of charge transfer between neighbouring hopping sites and the second term depends on the change in activation energy with modulation. In addition, the piezoresistance is inherently dependent on the nature of how distortions are distributed along the DNA molecular junction, d*z*/d*L*.

The experimentally measured spring constant for a 14 base pair DNA molecule is on the order of 0.2 N m^−1^ (refs 28, 29) while the weakest bonds of the thiol linker group, the gold atomic bonds nearest the molecule, have a spring constant of 8 N m^−1^ (refs 25, 26). This, as we discussed earlier, suggests that DNA is the softest portion of the molecular junction and the majority of the tip modulation is distributed across the DNA molecule. Indeed, experimental studies into the mechanical properties of DNA molecules show that the molecule can be mechanically stretched easily with atomic force microscopy^29^,^31^ and optical tweezers^32^,^33^. Previously, we were able to show that the mechanical properties of short (6–26 base pairs) single DNA molecular junctions prepared with the STM-BJ can be described by the analytical model proposed by de Gennes^20^,^34^. In this model, DNA is assumed to have a ‘ladder' structure in which the backbone (ladder sides) is stiffer than the hydrogen bonds between base pairs (ladder rungs). Using this model, de Gennes was able to show that the displacement between base pairs depends on the position along the strand as Δ*z*∝cos*h*(*χ*^*n*^), where *χ*^−1^ describes the number of base pairs affected by the stretching and *n* is the base pair position relative to the centre of the sequence. This way, stretching of the DNA molecules is unevenly distributed along the sequence, with bases at the end being strongly distorted while bases in the middle are only weakly affected. This model is further supported by force spectroscopy measurements by Hatch *et al*.^35^ showed that shear force is distributed over the ∼7 base pairs at the end of the DNA molecule. Thus, the model of de Gennes can be applied to the d*z*/d*L* expression in equation (3) to determine the change of displacement of the *n*th base pair with overall stretching length as


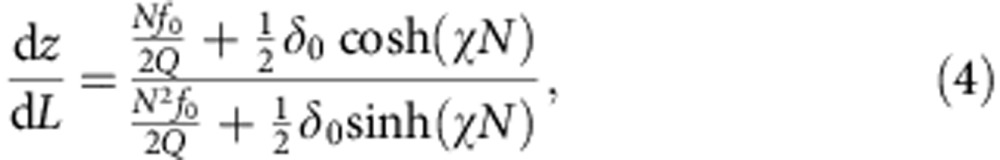


where *f*_0_ is the stretching force of the end base pairs, *Q* is the backbone spring constant and *δ*_0_ is the initial base pair separation (with fitting parameters of 4 pN, 30 N m^−1^ and 0.2 nm, respectively^34^,^35^,^36^).

Figure 4c,d shows the fitting of the measured piezoresistance to equation (3), utilizing the base to base transfer rate measured by Lewis *et al*.^37^ (*k*≅10^9^ s^−1^) and assuming that the contact transfer rate is much smaller (*k*_*L*_≅10^8^ s^−1^) because the contact resistance is larger for single DNA molecules^9^,^18^. We find that the transfer rate decay constant is larger for the intra-strand purine stacked sequence than for the inter-strand stacked purines sequence, *β*_G·G_=3.8±1.2 nm^−1^ and *β*_G·C_=1.3±0.3 nm^−1^, respectively. These decay constants are similar in magnitude to the decay constants measured for molecular systems with strong overlap between π-orbitals and gold electrodes, for example, 0.8 nm^−1^ measured by Diez-Perez *et al*.^2^ and 2 nm^−1^ measured by Meisner *et al*.^38^. This is a reasonable result considering the interaction between hopping sites is facilitated by π–π stacking between guanine bases. Furthermore, considering the spatial distribution of bases for intra-strand versus inter-strand sequences, Fig. 2a,b, it is straightforward to see that the more spatially overlapped sequences, intra-strand purines, will be more sensitive to mechanical modulation, resulting in a larger decay constant.

In addition to the charge transfer rate, the change in activation energy with modulation is dependent on the DNA sequence. According to the fitting with equation (3), the sequences with intra-strand purine stacking have a larger change in activation energy than inter-strand purines, d*E*_*BF*_/d*z*_G·G_=0.15±0.02 eV nm^−1^ and d*E*_*BF*_/d*z*_G·C_=0.07±0.006 eV nm^−1^, respectively. Sequence dependence of the change in activation energy suggests that the orbital energy of the end base pairs is more sensitive to modulation for sequences with greater π–π interaction, which is analogous to the dependence of semiconductor piezoresistivity to crystallographic orientation^39^. This is also in agreement with the observation of delocalization effects^18^ for intra-strand guanine sequences. Delocalization of the molecular orbital over many bases is known to have an effect on the orbital energy alignment, due to stronger coupling involved in delocalized sequences. Thus, it is not surprising that sequences with delocalized orbitals are more sensitive to perturbation of the molecular structure.

## Discussion

**Figure 5:**
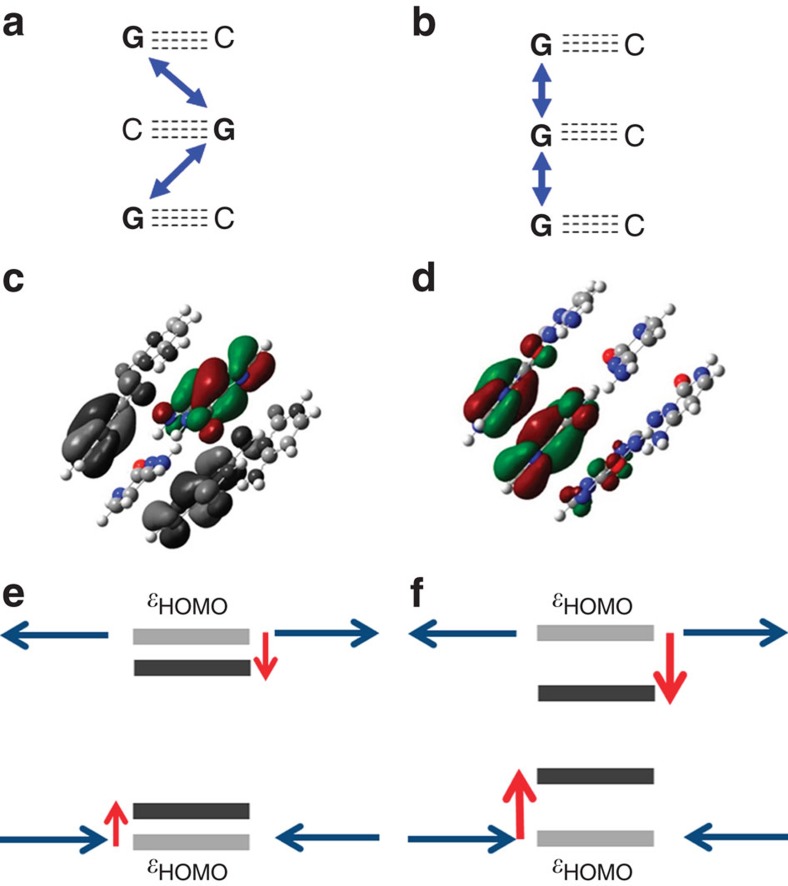
Schematic of calculation procedure. (**a**,**b**) 2D layout of base pairs calculated. Arrows indicate coupling between hopping sites. (**c**,**d**) Example of **CGC** and **GGG** sequence in B-form structure, respectively. The HOMO energy level of the centre base pair, in colour, is calculated. Note that grey scale orbitals in **c** are degenerate HOMO levels located on outside bases, while all green and red portions in **d** are the delocalized HOMO of the central base pair. (**e**,**f**) Demonstration of the HOMO energy level for the centre base pair increasing and decreasing with modulation. Intra-strand guanine sequence experiences larger amplitude shift in HOMO energy, and ultimately activation energy, with modulation.

For a deeper insight into this sequence dependence, we explored the effect of distance modulation on the site energy through semi-empirical quantum chemical calculations. Our model for the DNA molecule consists of three guanine–cytosine base pairs, either with guanines stacked (GGG) or with guanines alternating (CGC) between strands. Modulating the position of the first and third base pairs with respect to the middle base pair mimics the effect of stretching and compressing the DNA molecule, and allows us to probe the site energy of the middle base pair, as shown in Fig. 5. In agreement with the experimental fitting, we find that the change in energy with respect to modulation is larger for intra-strand guanines (GGG) than for inter-strand guanines (CGC). A similar trend is seen in Supplementary Fig. 6 for molecules containing adenine and thymine (AGA and TGT), showing that the site energy fluctuations induced by the mechanical modulation are different for intra-strand purine and inter-strand purine stacked sequences.

In summary, we demonstrate piezoresistivity in double helical DNA with different lengths and sequences. The piezoresistivity is larger for sequences with intra-strand purine stacking than for inter-strand purine sequences. By investigating the length dependence of both piezoresistance and conductance, we conclude that the DNA piezoresistance is determined by the sensitive dependence of the electronic coupling between neighbouring bases, along with the bridge site energy on mechanical force. In addition, we find similar delocalization effects as Xiang *et al*.^18^ for sequences with intra-strand stacked purines, which is closely related to the increased electronic coupling and change in hopping site energy. The thermally activated hopping model for charge transport, together with de Gennes DNA ladder model, fit the experimental data well to determine the relative importance of transfer rate and activation energy in the measured piezoresistance. Furthermore, our findings are supported by quantum mechanical calculations of the electronic couplings and site energy fluctuations. This work reveals important electromechanical functions of DNA, which is relevant to potential micro-electromechanical systems applications with DNA nanostructures, and also sheds light on the sensitive structural dependence of the charge transport of DNA on the π–π stacking interaction and hopping site energy of individual bases.

## Methods

### DNA sample preparation

The oligonucleotides used in this study were purchased from Integrated DNA Technologies (IDT), already having been high-performance liquid chromatography purified. To facilitate functionalization to a the gold electrodes, the oligonucleotides were purchased with a thiol linker (3′-thiol modifier c3 S–S) at the 3′ end, which is shipped and stored with a mercaptopropanol disulfide protection group. On receipt, the oligonucleotide samples were suspended in 18 MΩ deionized water to a desired concentration of 100 μM and stored at −20 °C. Before tip modulated (TM)-STM-BJ experiments, phosphate buffer solution (pH=7.0) was prepared with 100 mM Na^+^ and 10 mM *tris*(2-carboxyethyl)phosphine (TCEP) concentration, for deprotecting the thiol linker. The oligonucleotide sample to be studied was added to the TCEP solution at a concentration of 10 μM and allowed to react (deprotect) for ∼3 h at room temperature. The oligonucleotide sample was transferred to a spin column (Roche Applied Science quick spin column sephadex G-25) and centrifuged to remove TCEP and the protection group from the oligonucleotide sample. The oligonucleotide samples were then annealed by heating to 95 °C and gradually cooling to 4 °C over 4 h. The sample was then kept at 4 °C until the TM-STM measurements were performed. All TM-STM-BJ measurements were performed in phosphate buffer environment at room temperature. The structure of the oligonucleotides samples under experimental conditions was checked by circular dichroism, polyacrylamide gel electrophoresis, and melting temperature measurements, see Supplementary Figs. 7–9 and Supplementary Note 5 for details.

### TM-STM-BJ sample preparation and measurements

The STM substrates used in these experiments were prepared by thermally evaporating ∼1,300 Å of gold (99.9999% purity, Alfa Aesar) onto freshly cleaved mica slides and annealed in vacuum to produce Au(111) surfaces. Before TM-STM-BJ experiments, the substrate was flame annealed for ∼1 min with a hydrogen flame. STM tips used were produced by mechanically cutting gold wire (99.95% purity, Alfa Aesar) then coated with Apiezon wax to reduce leakage current during measurements in aqueous environment.

TM-STM-BJ experiments were performed using a Digital Instruments Nanoscope IIIA controller with a Molecular Imaging STM head and scanner. For all experiments, a current preamplifier with gain of 10 nA V^−1^ was used and the STM piezosensitivity in the *z* axis was 3.9 nm V^−1^, calibrated by measuring the height of Au atomic steps on Au(111) substrates. A custom designed Labview (National Instruments) program was used to control the STM scanner during TM-STM-BJ experiments and data collection. Molecular junctions were formed using the traditional break junction method, in which the tip was approached towards the surface until the amplifier saturation was achieved, then continued to approach for another 300 ms, at a ramp rate of 5 V s^−1^. A bias of 30 mV was applied to the substrate while the tip was held at virtual ground, the preamplifier input. The 5-mV amplitude, sinusoidal tip modulation at 1 kHz was applied using an external function generator (Stanford Research Systems) to the *z* axis piezo controller during the entire experiment. The modulation frequency was chosen to be well below the bandwidth of the current preamplifier (10 kHz) and tip piezos (3 kHz). The molecular plateaus in current were detected by an algorithm in Labview (National Instruments) and the current recorded while tip modulation was applied with the same program. The molecular piezoresistance was analysed in another home built Labview program, which filtered the current into a d.c. (<500 Hz) and tip modulation (between 900 and 1,100 Hz) portions for each molecular junction and combining these portions into a single 2D *α* versus conductance histogram for each molecular junction. Supplementary Fig. 1 shows the d.c. and tip modulation portions of the current for a molecular junction which is sustained for the 100ms measurement and a molecular junction which breaks down during the 100ms measurement, demonstrating the difference in conductance and \alpha with and without a molecule bridging the two electrodes. Histograms of individual junctions were added together to form 2D histograms for each molecular sequences studied (Supplementary Figs 2 and 3) and the piezoresistance and molecular conductance were determined by projecting a one-dimensional histogram onto the appropriate axis. Due to low yield of measurements, caused by low DNA concentration and short junction lifetime, each experiment yielded data from ∼50 molecular junctions. The experiments were then repeated until the total number of single molecule junctions was >400, and experimental data were compiled to form 2D histograms. The resulting one-dimensional conductance or piezoresistance histograms were then analysed using Origin 8.0 (OriginLab) with Gaussian or log-normal distributions, respectively, to determine the peak position and fitting error. All values displayed in this work are fitted peak positions and errors are fitting errors.

### Simulation structures and energy calculations

The effective electronic couplings were calculated considering the canonical double-stranded B-DNA conformation of the experimental sequences and under a two-state model and using the INDO/S Hamiltonian, which has been widely use for the surprising qualitative agreement in DNA calculations^21^. We removed the backbone and capped with Hydrogen atoms and for simplicity, we neglected the couplings of the end base pairs, although they were included to obtain the Hamiltonian in atomic orbitals. We calculated the effective coupling as the *V*_rms_, of the couplings along the sequences, in such way, we include the difference between *V*_GX_ and *V*_XG_ (X=C, T and A), in a similar manner as Berlin *et. al*^22^ To obtain the energy shift of the hopping site related to the change in the activation energy, we built a model consisting in three base pairs GGG and GCG using the canonical B-DNA structure, including the complementary sequence and omitting the backbone. We modulated the base pair–base pair distance to mimic the stretching and compressing effect from 3.2 to 3.8 Å, assuming that at such small experimental modulation, the ‘raise' parameter is representative of the complex dynamics that takes place. We calculate the HOMO energy of the middle base pair by block diagonalizing the Hamiltonian matrix corresponding of such fragment in the presence of the end base pairs.

## Additional information

**How to cite this article:** Bruot, C. *et al*. Piezoresistivity in single DNA molecules. *Nat. Commun.* 6:8032 doi: 10.1038/ncomms9032 (2015).

## Supplementary Material

Supplementary Information Supplementary Figures 1-9, Supplementary Tables 1-2, Supplementary Notes 1-5 and Supplementary References
