# Supplementary material for: Piezoresistivity in single DNA molecules
Source: Nat Commun. 2015 Sep 4;6:8032. doi: 10.1038/ncomms9032 (PMC4569700; doi:10.1038/ncomms9032)
Supplement: Supplementary Information — Supplementary Figures 1-9, Supplementary Tables 1-2, Supplementary Notes 1-5 and Supplementary References [file ncomms9032-s1.pdf]

Supplementary Information

Supplementary Figures

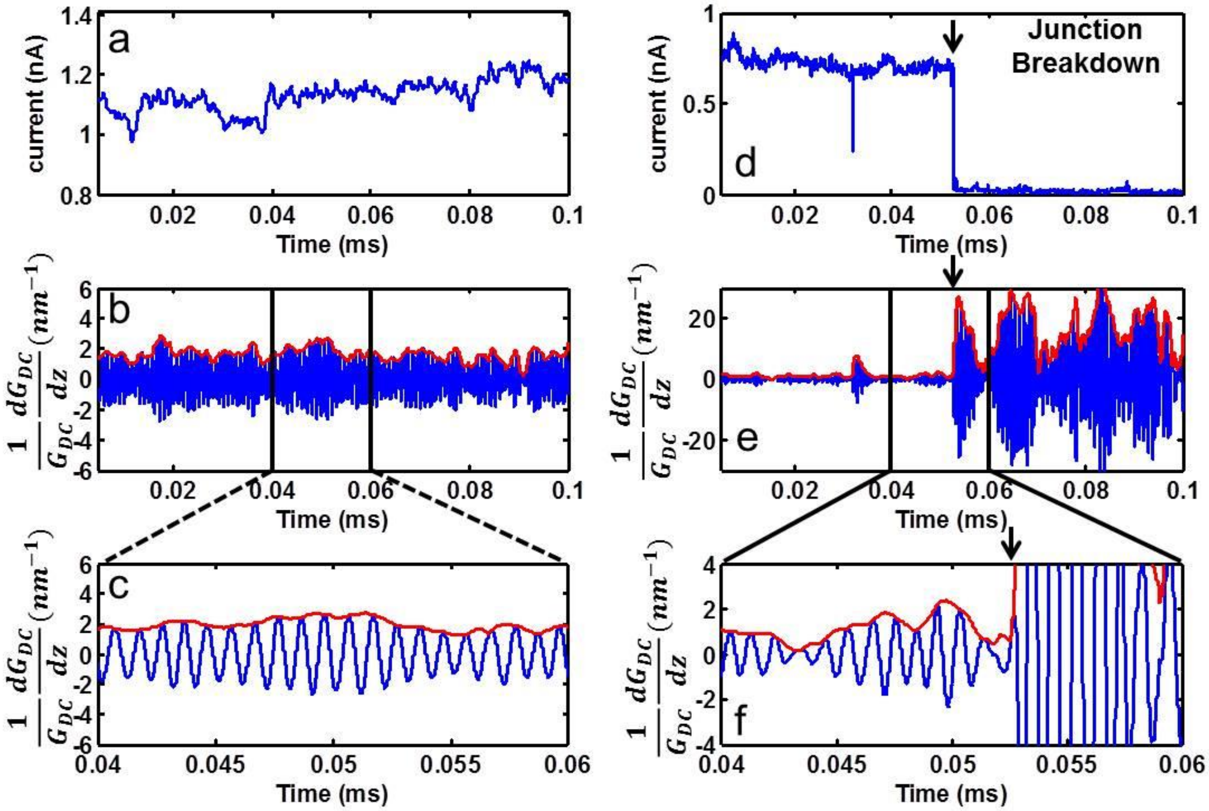

Supplementary Figure 1 | Tip modulated STM break junction measurement during junction breakdown. (a) DC current measurement during TM-STM break junction experiment in which molecular junction is sustained for 100ms. (b) $1/G_{DC}\, dG_{DC}/dz$ signal measured from the current signal at the tip modulation frequency. (c) Enlarged view if $1/G_{DC}\, dG_{DC}/dz$ signal to show the sinusoidal response in the current associated with the piezoresistance. (d) DC current of tip modulated STM break junction measurement in which the junction breaks down during the

100ms measurement. Break down is signaled by the black arrow. (e) Tip modulation frequency portion of current showing the large increase in signal after the molecular junction breakdown. (f) Enlarged view of (e) at the position of the breakdown.

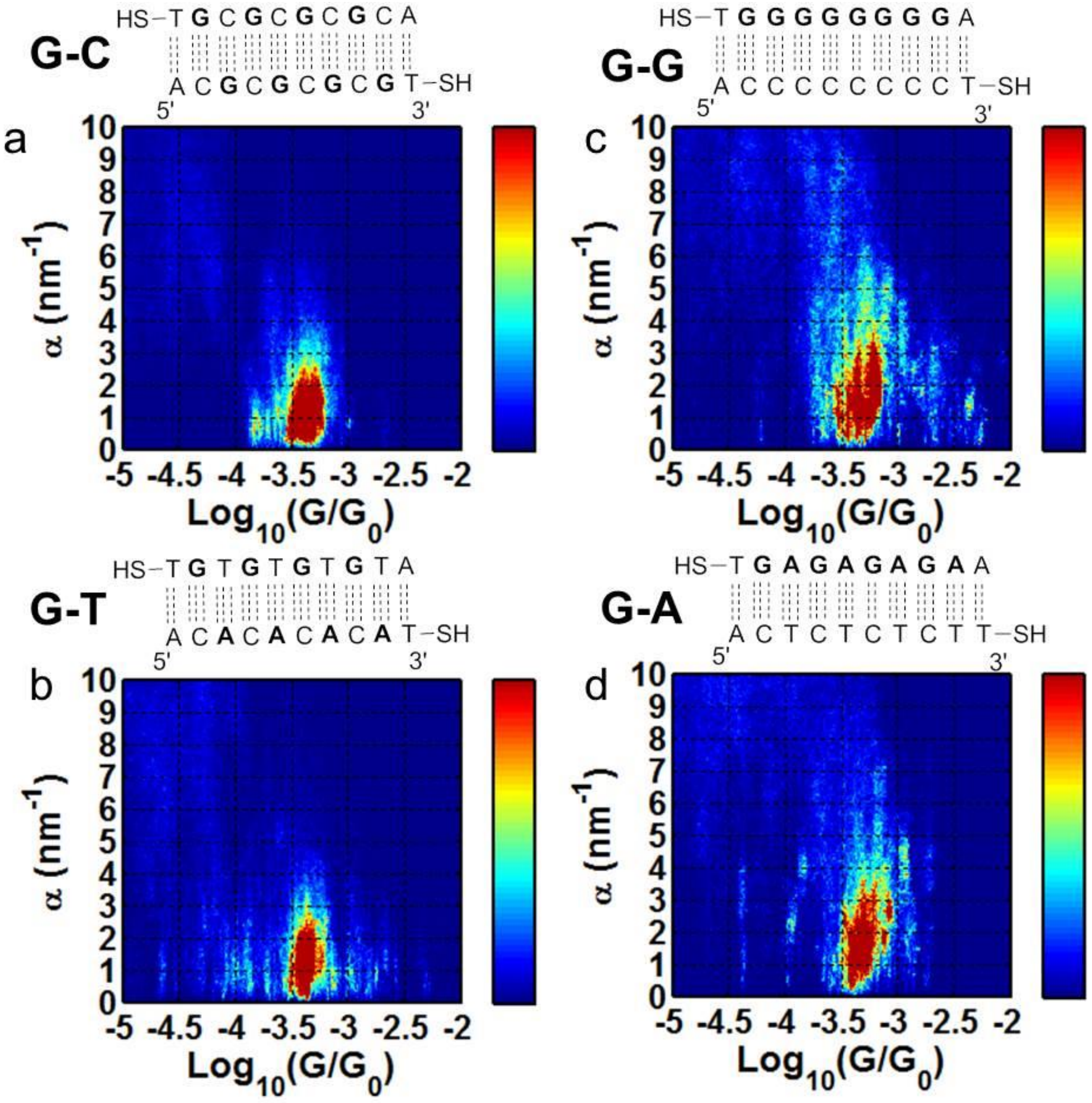

Supplementary Figure 2 | 2D α vs. conductance histograms for DNA molecules with different sequences studied.

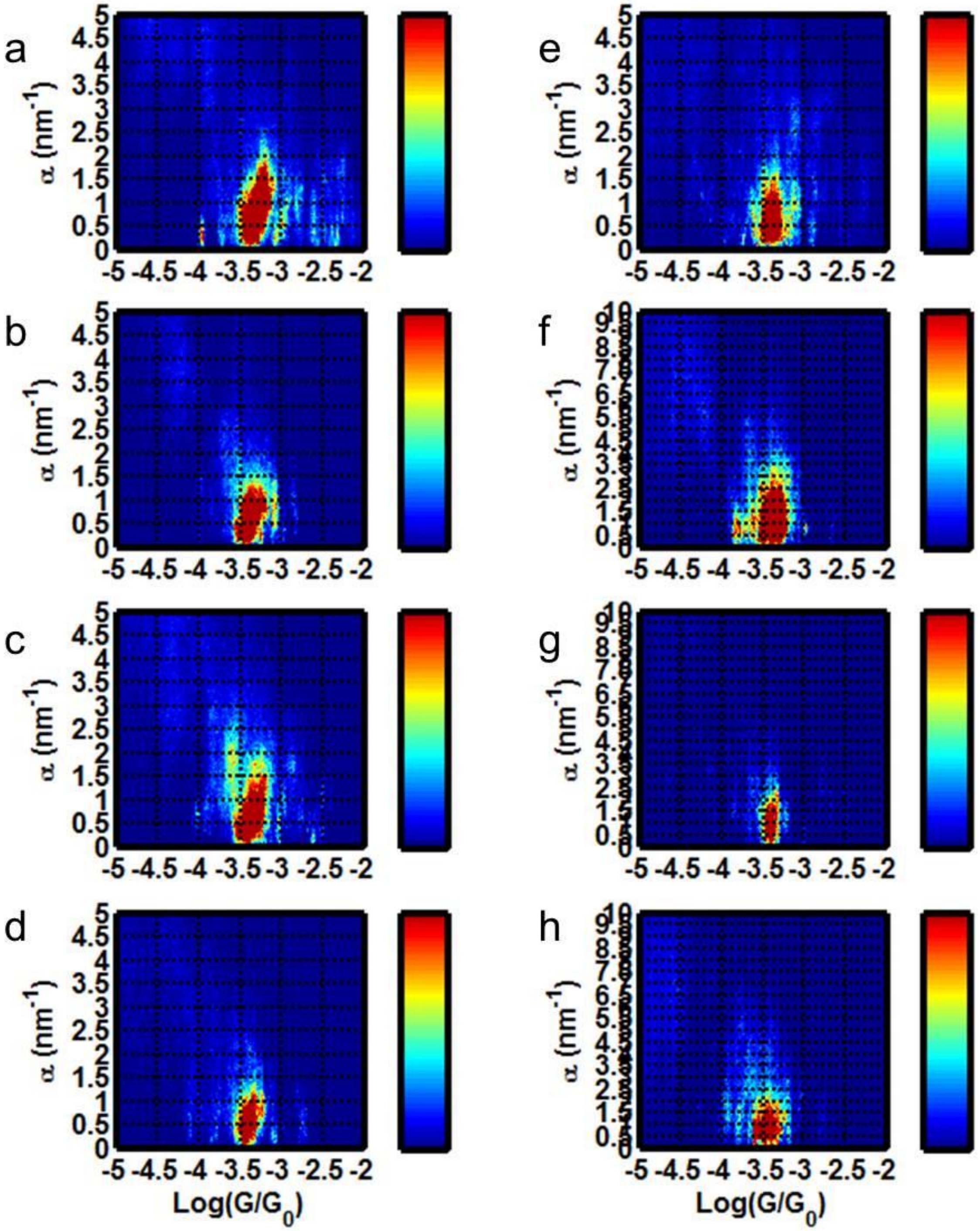

Supplementary Figure 3 | 2D α vs. conductance histograms for molecules of different lengths. (a-d) Histograms for $\mathbf{C_N}$-$\mathbf{G_N}$ (N=3,4,5,6 respectively). (e-h) histograms for $\mathbf{(C\text{-}G)_N}$ (N=3,4,5,6 respectively).

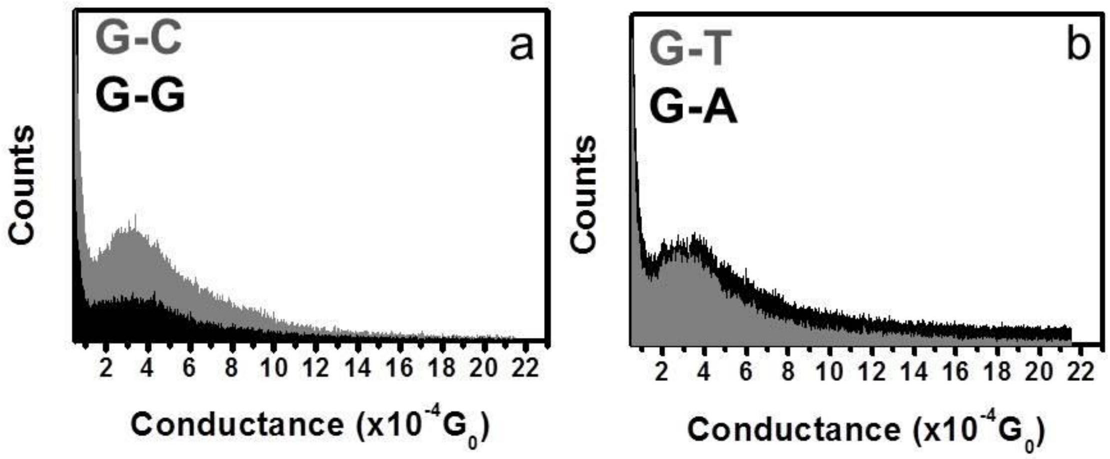

Supplementary Figure 4 | Linear conductance histograms for G-G, G-C, G-A and G-T sequences.

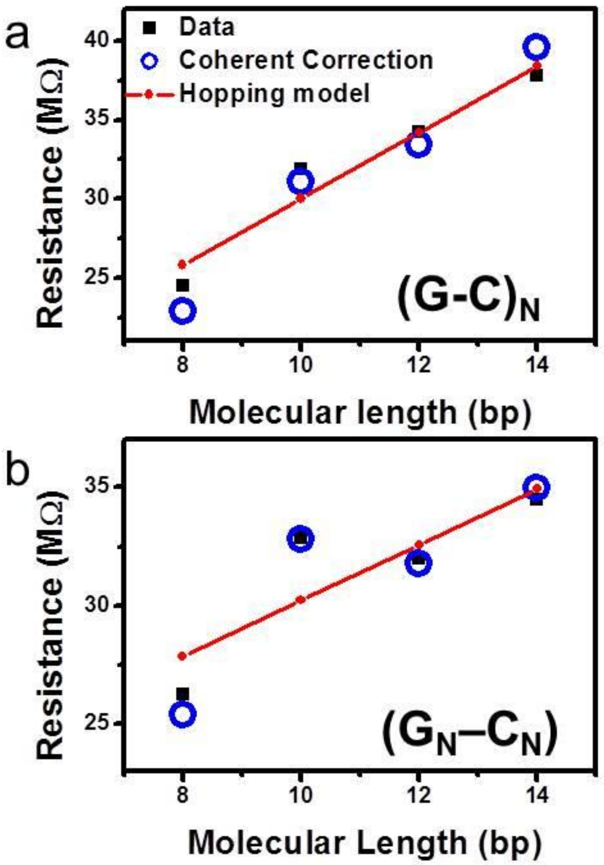

Supplementary Figure 5 | Resistance vs. molecular length fitting for inter-strand (a) and intra-strand (b) purine stacked molecules. Fitting these data (black squares) with the linear hopping

model (red filled circles connected by lines) and coherent correction to hopping model (blue open circles) we find that the inter-strand purine sequence is better fit with the linear hopping model while the intra-strand purine sequence is better fit with coherent corrected hopping model.

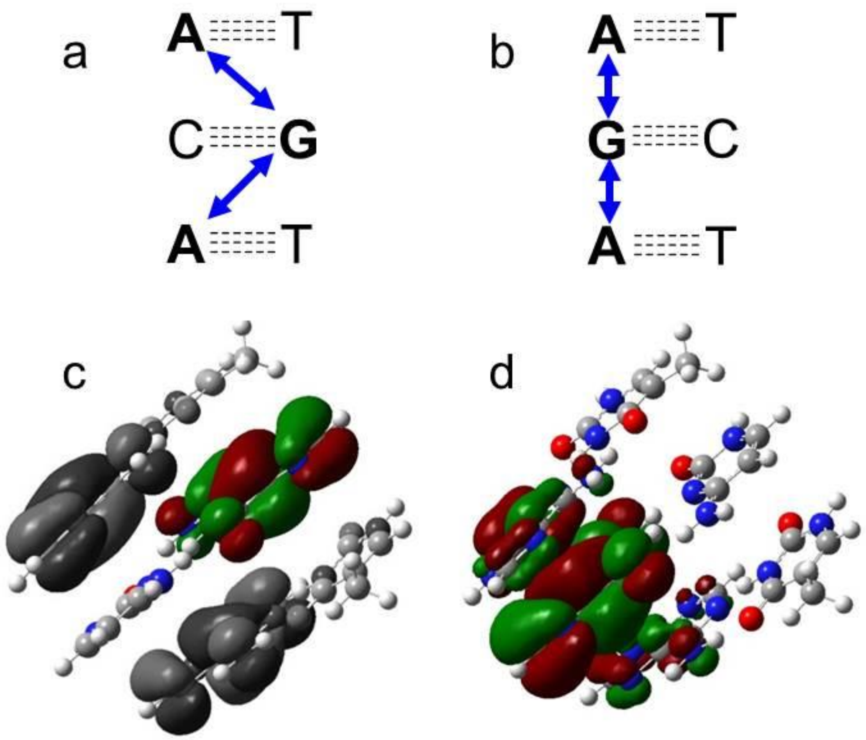

Supplementary Figure 6 | Calculated orbitals for neighboring adenine-thymine and guanine-cytosine base pair sequences. (a-b) Schematic illustrating the sequence and expected hopping pathway for TGT and AGA sequences, respectively. (c-d) HOMO orbitals of TGT and AGA, respectively, illustrating the distribution of hopping sites for inter- and intra-strand purine stacking. Gray scale orbitals in (c) are degenerate HOMO-1 orbitals located on adenine bases.

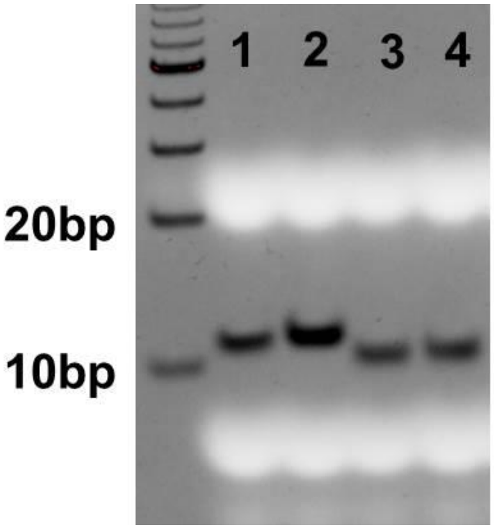

Supplementary Figure 7 | Native PAGE measurement. From left to right: 5’-A(AG)$_4$T-3’, 5’-A(TG)$_4$T-3’, 5’-A(AG)$_3$T-3’, 5’-A(C$_3$G$_3$)T-3’.

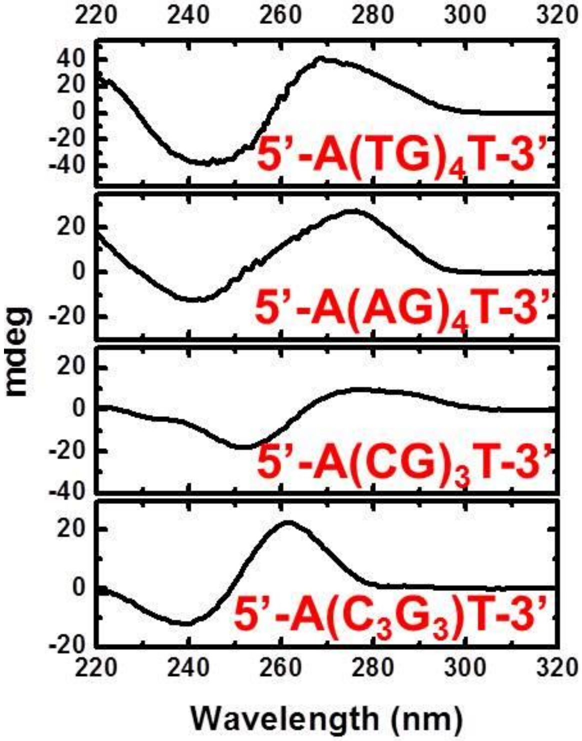

Supplementary Figure 8 | Circular dichroism spectra for DNA samples. From top to bottom: 5’-A(AG)$_4$T-3’, 5’-A(TG)$_4$T-3’, 5’-A(AG)$_3$T-3’, 5’-A(C$_3$G$_3$)T-3’

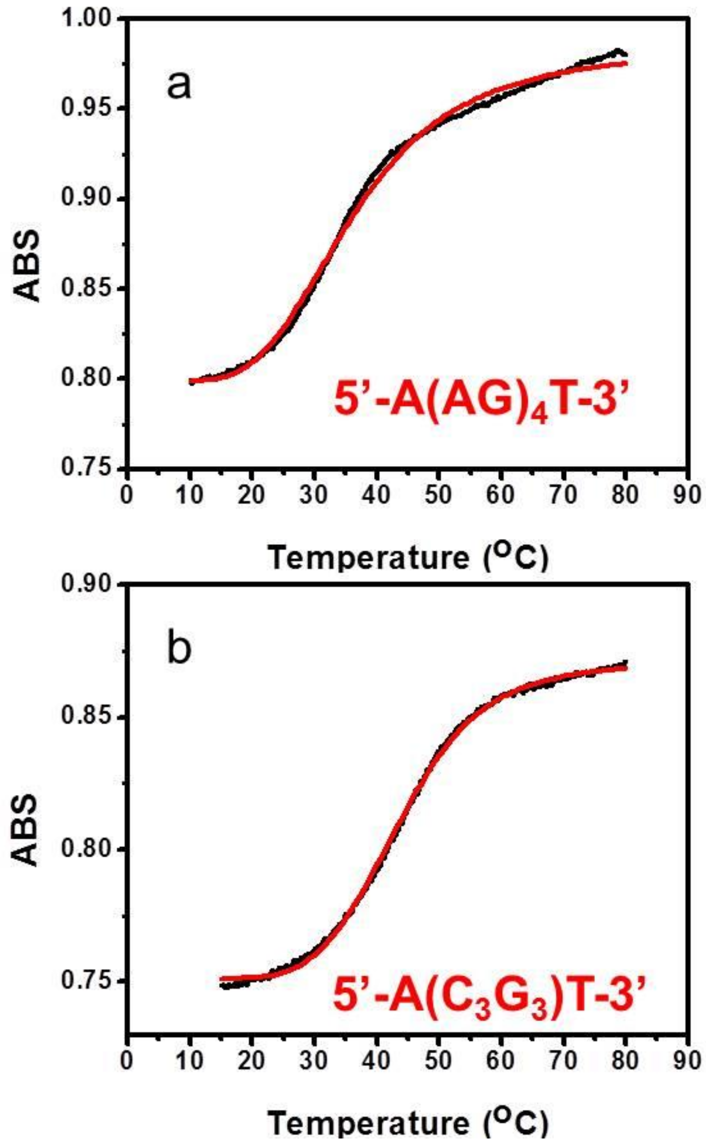

Supplementary Figure 9 | Melting temperature absorption curves for (a) 5’-A(AG)$_4$T-3’ and (b) 5’-A(C$_3$G$_3$)T-3’.

Supplementary Tables

Sequence	Conductance (x10^{-4} G_0)	Coupling (eV)	Piezoresistance (nm^{-1})	ΔE (eV)
Purine-Pyrimidine stacking				
**G-C**	4.05±0.03	0.107	0.85 ± 0.02	0.010
**G-T**	3.23 ± 0.04	0.076	0.92 ± 0.02	0.008
Purine-Purine stacking				
**G-G**	5.17 ± 0.07	0.144	1.35 ± 0.02	0.038
**G-A**	4.78 ± 0.02	0.115	1.45 ± 0.03	0.029

Supplementary Table 1 | Schematic of molecular structure, DC conductance, calculated couplings between neighboring sites, α values and calculated energy difference of the hopping site under ±0.01 nm mechanical modulation. Conductance and α values are peak position from 1-D histograms. Errors in conductance and α are fitting error.

X	V_{GX}(eV)	X	V_{XG}(eV)
C	0.052	C	0.042
T	0.0156	T	8.06x10^{-4}
G	0.141	G	0.141
A	0.13	A	2.13x10^{-3}

Supplementary Table 2 | Electronic couplings for dsB-DNA stacked base pairs from 3' to 5' for V_{GX} and V_{XG} where X= C, T, A, G.

Supplementary Notes

Supplementary Note 1 | Tip modulated STM break junction control experiments

Supplementary Figure 1 demonstrates that the measurements during the tip modulated STM break junction technique are indeed single molecule in nature. As described in the main

text, the tip retraction is stopped when a molecular plateau is detected in the current. The current is then monitored for 100ms while a sinusoidal modulation is applied to the tip position. During this process, due to the finite nature of single molecule junctions, the junction can spontaneously break down, resulting in a sudden decrease in current, to the amplifier background. Supplementary Figure 1 shows two separate molecular junctions, Supplementary Figure 1a and S1d, in which the tip modulated STM-BJ technique is used to measure piezoresistance. The junction in Supplementary Figure 1a-c shows a typical molecular junction in which the molecular plateau is maintained through the entire measurement. Supplementary Figure 1d-f, on the other hand shows a molecular junction which spontaneously breaks down during the tip modulated STM break junction measurement. The sudden decrease in current, which is associated with the breaking of a single molecule junction, is accompanied by a sudden increase in the derivative response ($1/G_{DC}\, dG_{DC}/dz$) which is a result of the change in transport mechanism from single molecule to tunneling through solvent. Considering the case when the molecular junction has broken down, the tip modulated STM measurement will be probing the tunneling decay constant of the solution environment, in this case phosphate buffer. The decay constant in these solvents have been shown to be on the range of ~10nm^{-1}, in agreement with the derivative signal after the breakdown (see Supplementary Figure 1e and f).

Supplementary Note 2 | Sequence dependent measured and calculated values

The measured conductance and piezoresistance values are presented below. The conductance values differ from those previously measured through STM break junction technique because of differing linker group and differing sequence. However, the order of magnitude is roughly similar and the differences are predictable. For example, the present conductance is smaller than that of Xu et al., which is understandable by the difference in

sequence between these two experiments. Particularly, the addition of an A-T base pair at the ends is expected to decrease molecular conductance. Additionally, the present conductance is smaller than that of Xiang et al., who use very similar sequences, but use modified thiamine bases to directly contact the DNA base pairs to the electrodes, while the present work uses thiol linkers contacting the phosphate backbone. This difference in linker group is expected to increase the number of bonds between bases and electrode, thus increasing contact resistance.

Supplementary Note 3 | Coherent correction fitting

Following the method of Xiang et al.[1] we modeled transport in the inter-strand and intra-strand purine stacked sequences of Figure 4 in an intermediate regime, between coherent tunneling and thermally activated hopping, in which the hopping site is delocalized over several neighboring guanine bases. To achieve this we used the coherent correction to the hopping model developed by Büttiker[2]. In this model the resistance of a DNA molecule can be expressed as

$$R_{tot} = R_0 + \frac{h}{e^2}\frac{N-1}{1-2e^{-B(N-1)}\cos[C(N-1)]}T_{GG-st}^{-1}. \qquad (1)$$

Here the first term (R_0) is the contact resistance, which is determined using the linear fitting of the inter-strand purine sequence, and the second term is the coherent corrected hopping through the intra-strand purine sequence. The fitting parameters include T_{GG-st}^{-1} the transmission probability between neighboring guanines, $B = \omega_0/v\tau_i$ which reflects the coherence length for inelastic scattering on a hopping site (i.e. the size of the hopping site), and $C = \frac{2\sqrt{2mE}}{\hbar}\omega_0$ which gives the hole mass and energy, *m* and *E* respectively. To use the coherent correction, first a linear fitting of the resistance vs. molecular length was used to determine the y-intercept, the value of R_0 above, for both inter-strand and intra-strand purine sequences (R_0 = 13.1 MΩ and 20.7 MΩ respectively). Next the coherent correction was used to fit the resistance vs. molecular

length data for both sequences. The fitting parameters for the inter-strand sequence were T_{GG-st}^{-1} = 5.29±0.38 MΩ/bp, B = 0.923±0.38 nm, and C = 4.06±0.43eV. The fitting parameters for the intra-strand sequence were: T_{GG-st}^{-1} = 2.95±0.14 MΩ/bp, B = 0.67±0.08 nm, and C = 2.09±0.14eV. As can be seen in Figure S4, comparison of the linear (pure hopping) and coherent correction fitting sheds light on the most appropriate model for each sequence. One way to quantify this is to consider the R^2 value obtained from fitting to each model. For the inter-strand purine stacked sequence (Supplementary Figure 4a) fitting with the hopping model (linear fitting) resulted in R^2 = 0.910 while fitting with the coherent correction resulted in R^2 = 0.759, suggesting that the most appropriate model for transport in inter-strand purine stacked sequences is hopping between neighboring guanines. Alternatively, fitting the intra-strand purine stacked sequence to the hopping model resulted in R^2 = 0.599 while fitting to the coherent correction resulted in R^2 = 0.914, supporting the previously observed delocalization in stacked guanine sequences and suggesting that there is a coherent portion of the hopping transport.

In addition to indicating that hopping in intra-strand stacked sequences involves delocalized, coherent hopping sites, the fitting data above also supports the assumption of small contact transfer rate compared to the base to base transfer rate. We can see this by comparing the contact resistance (R_0 = 13.1 MΩ and 20.7 MΩ for inter-stand and intra-strand respectively) with the slope of molecular resistance vs. molecular length (2.36 and 4.21 MΩ/bp respectively) that the limiting factor in transport through DNA molecular junctions is the contact resistance. This observation has been seen previously and the present resistance vs. length decay are similar to those of Xu et al.[3] (~11 MΩ/bp) and Xiang et al.[1] (~0.57 MΩ/bp).

Supplementary Note 4 | Guanine-adenine interaction

It has been reported earlier that addition of multiple adenine-thymine base pairs between guanine-cytosine tracts creates a tunneling barrier[3]. This tunneling barrier is seen as an exponential length dependence of conductance with the addition of adenine-thymine base pairs, in multiples of 2. However, as we note in the main text, the addition of individual adenine-thymine base pairs, between guanine-cytosine base pairs, does not appear to substantially alter the conductance or hopping transport mechanism of single DNA molecules. This is seen when comparing the conductance values in Supplementary Table 1. We attribute this observation to the similarity in coupling between neighboring guanine-cytosine base pairs and neighboring guanine-cytosine and adenine-thymine base pairs. As can be seen in Supplementary Table 2, the calculated coupling between neighboring guanine and adenine bases can be of the same order of magnitude as that between neighboring guanine bases, depending on the order of the sequence. Similar observations can be made about the coupling between neighboring guanine and thymine bases. To further illustrate this, Supplementary Figure 6, shows the calculated highest occupied molecular orbitals (HOMO) of a guanine-cytosine base pair surrounded by adenine-thymine base pairs, in either TGT or AGA order. When purines are stacked inter-strand (TGT) the HOMO is isolated on the guanine, while the HOMO-1 is degenerate on the adenine bases due to mismatch in orbital energy of guanine and adenine. However, for intra-strand stacked purine sequences (AGA), the HOMO is delocalized from the central guanine base to partially reside on the neighboring adenines. This is a similar effect as that seen for neighboring guanine bases, as discussed in Figure 5 and in the main text.

Supplementary Note 5 | DNA sample structure characterization

In order to ensure that the DNA samples measured were indeed double helical B-form DNA during the experiments, we performed circular dichroism (CD), native polyacrylamide gel

electrophoresis (PAGE) and melting temperature measurements. These measurements were performed under the same buffer concentrations as the tip modulated STM-BJ experiments, and when applicable at room temperature, in order to fully characterize the structure of the DNA molecules during the break junction experiments.

PAGE:

Double strand DNA structure was confirmed by native polyacrylamide gel electrophoresis (PAGE). PAGE measurements were performed at 20°C with a voltage of 200V for approximately 3 hours using 50 pMol of each sample and 12% native PAGE gels in 1× TAE Mg^{2+} buffer. Ethidium bromide (EB) was used to stain the gels and a Biorad Gel Doc XR+ system was used to for sample visualization. Supplementary Figure 7 shows the DNA samples measured with PAGE annealed in 100mM Na^{+} phosphate buffer solution. The samples measured are 10 (1 and 2) and 8 (3 and 4) base pairs long and are intended to represent the measured samples which are least stable at room temperature. The presence of a single band in the range of 10bp indicates that the samples are double stranded hybridized DNA structure.

Circular dichroism measurments:

Circular dichroism measurements of the DNA samples were used to confirm the B-form structure of the DNA samples measured. The CD measurements were performed in a Jasco (Easton, MD) J-815 Spectropolarimeter from 320 nm to 220 nm with a scanning rate of 50 nm/min to determine the structure of DNA samples. The spectra were average results from 5 scans, taken in 100mM Na^{+} phosphate buffer solution with 5 uM of dsDNA at room temperature to mimic STM-BJ experimental conditions. The spectra, Supplementary Figure 8, are in good agreement with previously reported spectra of similar sequences[4–6]. Notably, inter-strand purine sequences are in exact B-form structure, while intra-strand purine sequences have subtle

structural changes from increased coupling between neighboring bases which result in slightly modified B-form structures.

Melting temperature:

Melting temperature experiments were performed in a Varian Cary 300 Bio UV spectrophotometer with a Peltier thermal controller to determine melting temperature. 5 uM dsDNA were prepared with 100mM Na^+ phosphate buffer and annealed as for STM-BJ measurements, then heated at a rate of 0.2℃/min from 10℃ to 80℃ with the absorbance at 260 nm recorded in 60s intervals. The samples measured, shown in Supplementary Figure 9, were initially predicted to have the lowest melting temperature, being shortest samples measured with guanine base content. Fitting the melting temperature curves to a two state thermodynamic model yielded a melting temperature of 36±0.69 ℃ for 5'-A$(AG)_4$T-3' and 43±0.48℃ for 5'-A(C_3G_3)T-3' sequences, well above the experimental temperatures of 22℃.

Supplementary References

1. Xiang, L. *et al.* Intermediate tunnelling–hopping regime in DNA charge transport. *Nat. Chem.* **7,** 221–226 (2015).

2. Buttiker, M. Coherent and sequential tunneling in series barriers. *IBM J. Res. Dev.* **32,** 63–75 (1988).

3. Xu, B., Zhang, P., Li, X. & Tao, N. Direct Conductance Measurement of Single DNA Molecules in Aqueous Solution. *Nano Lett.* **4,** 1105–1108 (2004).

4. Vorlickova, M., Kejnovska, I., Bednarova, K., Renciuk, D. & Kypr, J. Circular Dichroism Spectroscopy of DNA: From Duplexes to Quadruplexes. *Chirality* **24,** 691–698 (2012).

5. Ng, H.-L. & Dickerson, R. E. Mediation of the A/B-DNA helix transition by G-tracts in the crystal structure of duplex CATGGGCCCATG. *Nucleic Acids Res.* **30,** 4061–4067 (2002).

6. Kypr, J., Kejnovská, I., Renčiuk, D. & Vorlíčková, M. Circular dichroism and conformational polymorphism of DNA. *Nucleic Acids Res.* **37,** 1713–1725 (2009).
